# Functionalized Thick Film Impedance Sensors for Use in In Vitro Cell Culture

**DOI:** 10.3390/bios8020037

**Published:** 2018-04-05

**Authors:** Heike Bartsch, Martin Baca, Uta Fernekorn, Jens Müller, Andreas Schober, Hartmut Witte

**Affiliations:** 1 Electronics Technology Group, Technische Universität Ilmenau, IMN MacroNano^®^, 98693 Ilmenau, Germany; jens.mueller@tu-ilmenau.de; 2 Nano-Biosystems Technology Group, Technische Universität Ilmenau, IMN MacroNano^®^, 98693 Ilmenau, Germany; baca.martin2@gmail.com (M.B.); uta.fernekorn@web.de (U.F.); andreas.schober@tu-ilmenau.de (A.S.); 3 Institut de Neuroscience des Systèmes (INS), Aix-Marseille Université, 27 Bd Jean Moulin, CEDEX 13385 Marseille, France; 4 Biomechatronics Group, Technische Universität Ilmenau, IMN MacroNano^®^, 98693 Ilmenau, Germany; hartmut.witte@tu-ilmenau.de

**Keywords:** thick film sensors, impedance measurement, low temperature co-fired ceramics, functionalization, electrophysiological recordings

## Abstract

Multi-electrode arrays find application in electrophysiological recordings. The quality of the captured signals depends on the interfacial contact between electrogenic cells and the electronic system. Therefore, it requires reliable low-impedance electrodes. Low-temperature cofired ceramic technology offers a suitable platform for rapid prototyping of biological reactors and can provide both stable fluid supply and integrated bio-hardware interfaces for recordings in electrogenic cell cultures. The 3D assembly of thick film gold electrodes in in vitro bio-reactors has been demonstrated for neuronal recordings. However, especially when dimensions become small, their performance varies strongly. This work investigates the influence of different coatings on thick film gold electrodes with regard to their influence on impedance behavior. PEDOT:PSS layer, titanium oxynitride and laminin coatings are deposited on LTCC gold electrodes using different 2D and 3D MEA chip designs. Their impedance characteristics are compared and discussed. Titanium oxynitride layers emerged as suitable functionalization. Small 86-µm-electrodes have a serial resistance R_s_ of 32 kOhm and serial capacitance C_s_ of 4.1 pF at 1 kHz. Thick film gold electrodes with such coatings are thus qualified for signal recording in 3-dimensional in vitro cell cultures.

## 1. Introduction

**Figure 1:**
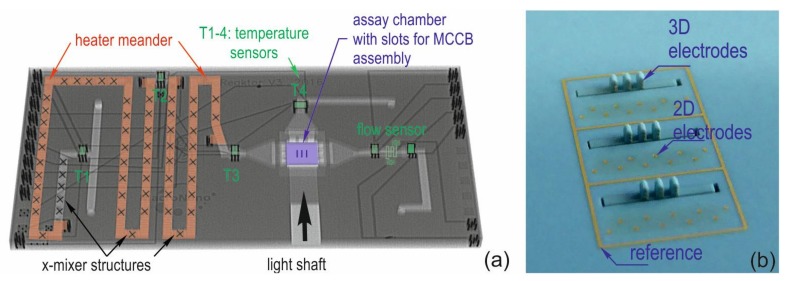
(**a**) Ceramic bio-reactor. The colored X-ray image makes it possible to view the integrated mixer, temperature control and flow monitoring. The base plate consists of glass and is mounted with epoxy at the bottom, next device generation with integrated 3D sensors is equipped with a ceramic base plate and slots for ceramic contact board assembly, indicated by the blue lines; (**b**) three miniaturized ceramic circuit boards, assembled perpendicular through slots of the ceramic base plate (LTCC test chip).

Brain-on-a-chip fluid systems must provide an adequate environment to support the growth of neuronal cells in three-dimensional hydrogels [1]. This encompasses a reliable fluid supply, provision of a nutrient supply at a constant temperature, and the reliable removal of metabolic products. Modern microfabrication offers a wide range of technologies, which enables the design of bio-microreactors [2]. Low-temperature cofired ceramic technology (LTCC) is suitable for the rapid prototyping of microreactors with integrated electronics [3,4,5]. LTCC reactors are steadily entering the field of bio-processing [5,6,7,8,9] and can monitor cell cultures [10]. The current work contributes to the 3D integration of sensor electrodes in such reactors. A prototype of an LTCC bio-reactor is depicted in Figure 1a.

It contains an x-mixer structure [11] and two independent heater elements, which regulated the nominal temperature of 37 °C with an accuracy of 0.2 K [8]. The flow sensor has a linear characteristic up to a flow rate of 80 µL/min [12]. A light shaft allows the lateral illumination of the assay chamber, which can carry 3D assays up to 2 mm in height. The current version is equipped with a glass base plate, which allows optical inspection trough the bottom. In the next device generation, this glass bottom is substituted by a co-fired ceramic bottom, which contains the slots for 3D sensor assembly (illustrated in Figure 1a), similar to the test chip in Figure 1b. Miniaturized ceramic contact boards can be mounted perpendicular through these slots in the base plate using wire bonding and epoxy sealing. This integration approach enables the 3-dimensional investigation of cell cultures of several hundred micrometers in height and justifies our motivation to study thick film electrode characteristics with regard to their application as impedance sensor elements.

The perpendicular assembly of miniaturized ceramic circuit boards (MCCB) on LTCC chips that carry thick film gold electrodes for capturing of neuronal signals has been the topic of previous work [13]. Over a period of six months, primary rat hippocampal and cortical cell cultures were monitored. The experience taught us that thick film gold electrodes are prone to surface adsorbates. These can affect the transition performance of the sensors. Plasma treatment [14] and wet chemical cleaning [15] can improve the performance.

Pursuing these studies, the present work investigates different influences on the impedance performance of thick film electrodes. 2D chips are used for the evaluation of functionalization layers and 3D chips for the assessment of protein coatings. This paper thus provides an orientation for future integration of 3D impedance sensor electrodes into LTCC bio-reactors.

## 2. Materials and Methods

### 2.1. Test Chip Types

**Figure 2:**
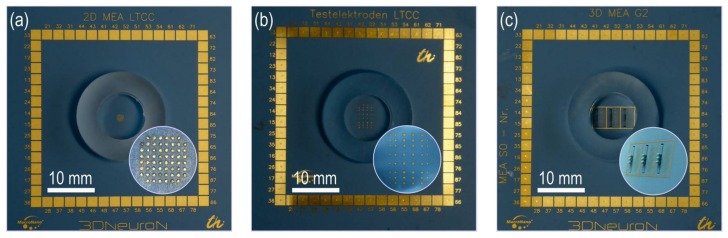
Test chip types (**a**) 2D test chip Type A; (**b**) 2D test chip Type B; (**c**) 3D test chip Type C. Number and size of electrodes is summarized in Table 1 for all types.

The current investigations use LTCC 2D and 3D test chips containing different multi-electrode array (MEA) designs. The impedance characteristic of electrodes and the influence of the functionalization layer on it can be investigated using 2D chips. In this way, assembly effort is reduced. Two different 2D-MEA layouts are available. The use of 3D chips is mandatory for the evaluation of cell culture preparation steps. The layout of the landing pads is compatible with the pad design of commercial recording systems (MEA2100, multichannel systems MSC GmbH, Reutlingen, Germany). The used test chip types are depicted in Figure 2, and the designs are summarized in Table 1. The 2D chips and 3D base plate are made of 4 layers of Green Tape™ DP 951 PX (DuPont Nemours, Wilmington, DE, USA). The electrodes are screen printed using the gold metallization DP 5740A (DuPont Nemours). Buried wirings and landing pads are screen printed using the same paste. For 3D MEAs, a miniaturized ceramic contact board (MCCB) is prepared using 4 layers of Green Tape™ DP 951 PT (DuPont Nemours). Electrodes and wiring are screen printed with the gold paste DP 5740A. The contour is laser cut in the green state. After firing, the batch has a thickness of 400 µm. The single MCCB elements are separated by dicing. 3D chips carry three of these MCCB, assembled through slots, which are laser cut into a 2D LTCC base plate. Wire bonds attached on landing pads at its backside contact the sawed metal surface through the via. Remaining gaps between MCCB and base plate are sealed with epoxy resin (HIE, IKTZ GmbH, Jena, Germany). The 3D assembly process is explained in the additional video. A fused silica ring with an inner diameter of 11 mm and a height of 7 mm is glued around the multi-electrode area using PDMS (Silguard 184) in order to provide a reservoir for the cell culture medium.

### 2.2. Test Procedure

#### 2.2.1. Cleaning

**Figure 3:**
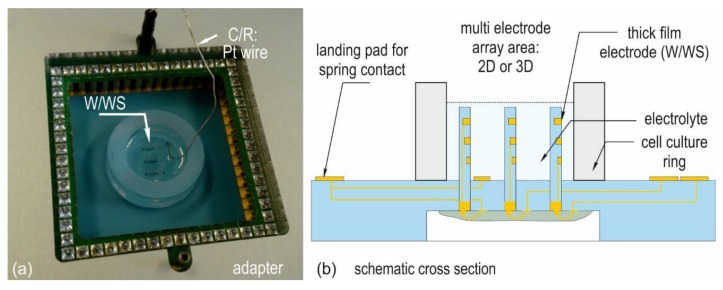
(**a**) LTCC chip mounted in the impedance measurement adapter; (**b**) cross section of the measurement set-up.

**Figure 4:**
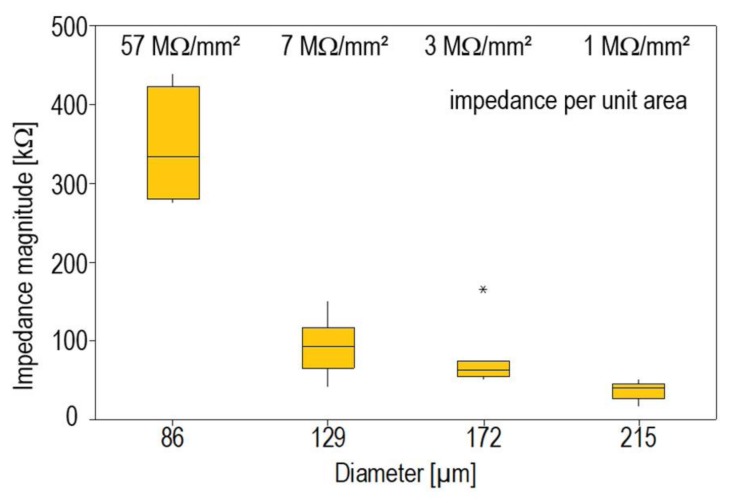
Impedance magnitude as a function of the electrode diameter of thick film gold electrodes, measured at a frequency of 1 kHz (Type B chip).

All samples were cleaned for 20 min in acetone, 20 min in isopropanol, and finally rinsed 5 min in distilled water. Drying was carried out under nitrogen flow using an air blow gun. Subsequently, the chips were treated in oxygen plasma for 5 min at 200 W. The application of PEDOT:PSS and laminin coating started within the first hour after cleaning. For TiO_x_N_y_ sputter deposition, the samples were transferred immediately from the plasma cleaning plant to the vacuum chamber of the sputter plant. The coated chips are measured using the set-up in Figure 3 and the method described in Section 2.3. Figure 4 depicts the results obtained from uncoated thick film gold electrodes after cleaning.

#### 2.2.2. Laminin Coating

**Figure 5:**
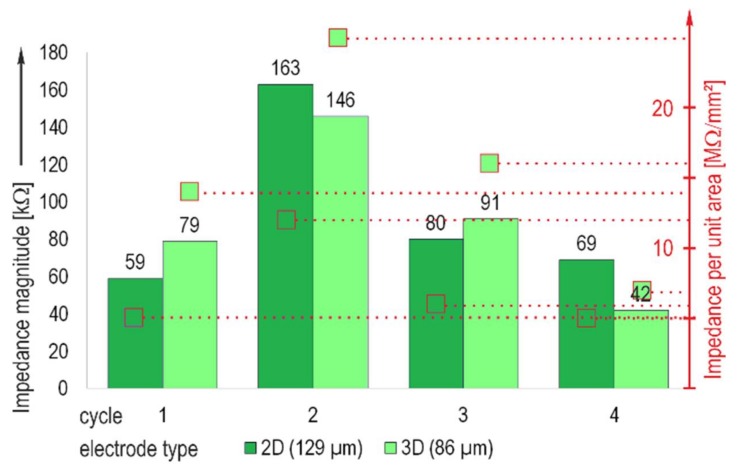
Median of the electrode impedance magnitude and impedance per unit area, measured at 1 kHz for the type C MEA, thick film gold with laminin coating.

Unlike the TiO_x_N_y_ and PEDOT-PSS functionalization, which is applied on 2D surfaces, laminin is coated before cell cultivation and must be pipetted on the sterilized chips before cell seeding. This extracellular matrix protein is often used to cover inorganic surfaces and provide a suitable environment for in vitro cell cultures, since it has a positive influence on cell adhesion. However, additional layers can attenuate the signal transmission at the bio-MEA interface [16]. Therefore, impedance changes after coating and repeatability after multiple applications must be evaluated. One chip type C underwent repeated coating and cleaning steps. Electrode performance and chip assembly were investigated. Figure 5 depicts the results of the impedance measurement, performed after each cycle. One cycle includes the following steps, which are carried out under sterile conditions in a laminar flow hood: before coating, the multi-electrode arrays are cleaned for 15 min with 70% ethanol, followed by a rinsing step with distilled water and air-drying under sterile conditions. Subsequently, a poly(ethyleneimine) (PEI) solution in distilled water (PEI, 0.05% *w*/*v*, Sigma Aldrich, Taufkirchen, Germany) is pipetted onto the MEA and incubated for 1 h at room temperature. After removing the PEI solution, the MEA is rinsed four times with distilled water and dried. The laminin coating solution consists of 100 mL laminin (Sigma Aldrich, St. Louis, MO, USA) pipetted into 5 mL sterile buffered saline solution. The solution is dispensed on the MEA. After an incubation time of 1 h at room temperature, the solution is removed using a pipette, and the MEA is immediately filled with cell culture medium 10% DMEM (Dulbecco’s Modified Eagle’s Medium, blended with 10% fetal calf serum, 100 U/mL penicillin, 100 μg/mL streptomycin).

Tergazym cleaning ensures the removal of residues after cell culture experiments. Multiple use of chips therefore requires various cycles of coating and tergazym cleaning. The solution contains 1 g Tergazym (ALCONOX/Z273287-1EA, Sigma Alsrich, St. Louis, MO, USA) dissolved in 100 mL distilled water. The whole chips are stored in the solution overnight at room temperature and they are carefully rinsed with distilled water. Drying is carried out under nitrogen flow using an air blow gun.

#### 2.2.3. Functionalization with Printed PEDOT:PSS

This functionalization was applied on three 2D chips of type A. The aqueous dispersion Clevios^TM^ PH 1000 (Heraeus, Hanau, Germany) was applied with a doctor blade on the thick film gold electrodes. The non-ionic surfactant Triton^TM^ X-100 (Sigma Alsrich, St. Louis, MO, USA) was added in two trials as wetting agent with a concentration of 0.01%. Drying temperature and blade gap were varied as additional parameters (see Table 2). All layers were cured at 200 °C for 30 min in a lab furnace, the ramp time for both, heating and cooling down was 30 min.

**Figure 6:**
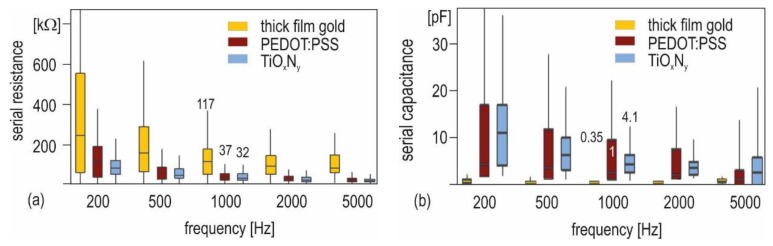
Serial equivalent circuit components as a function of the frequency; (**a**) serial resistance; (**b**) serial capacitance. Measurements on 86-µm electrodes are compared, including thick film gold, MEA type C; PEDOT:PSS coating on thick film gold, MEA type A and TiO_x_N_y_ coating on thick film gold, MEA type A.

**Figure 7:**
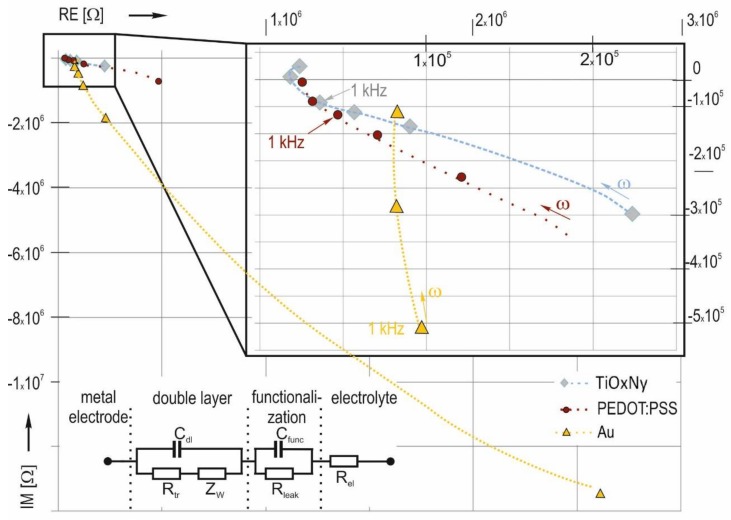
Nyquist plot for thick film gold electrodes, pure and coated with PEDOT:PSS and TiO_x_N_y_. The data points represent the median values of all measurements, captured on the test chips: PEDOT:PSS and TiO_x_N_y_ MEA type A, MEA type C. The equivalent circuit encompasses: R_el_—electrolyte resistance; C_dl_—double layer capacitance; R_tr_—charge transfer resistance; Z_W_—Warburg impedance; C_func_—functional layer capacitance; R_leak_—functional layer leak resistance.

Independent of wetting agent content, the dispersion wets the electrodes well. The impedance of the chips at 1 kHz was compared. Only the blade gap has been identified as the main influence factor on the resulting thickness of the layer, resulting in a remarkable effect on the electrical characteristics. The values of three independent measurements on sample 22 are represented in Figure 6. The respective median of all measured values is depicted as a Nyquist plot in Figure 7.

#### 2.2.4. Functionalization with Sputtered Titanium Oxynitride

Three test chips type A were used to evaluate the influence of titanium oxynitride (TiO_x_N_y_) coatings. The layers are sputtered on the thick film gold electrodes in a RF plasma recipient, applying 200 W for 2 min in an atmosphere of 80 Sccm nitrogen and 0.5 Sccm oxygen at a total process pressure of 1.3 mbar. A silicon reference chip with dimensions of 10 mm × 10 mm, half covered with a photo resist, is sputtered in the same process. The stripped border of this chip is measured in order to obtain the sputtered layer thickness. The thickness of the films is 25 nm. A box plot of serial resistance and serial capacitance is presented in Figure 6a,b, and the median of all values is depicted as Nyquist plot in Figure 7.

### 2.3. Impedance Measurement

A commercially available IC-chip for high-impedance biopotential monitoring is used for the impedance measurements (RHD213, Intan Technologies LLC, Los Angeles, CA, USA). The chip design encompasses the acquisition system for electrophysiological signals, arrays of low-noise amplifiers with analogue front-end, a multiplexed 16-bit analogue-to-digital converter, a flexible electrode impedance measurement module and a digital serial bus interface.

The printed circuit board contains two of these RHD2132 and connects the LTCC chip with 60 spring loaded contacts. It is connected with an USB/FPGA interface (Opal Kelly XEM6010, Opal Kelly, Portland, OR, USA), which links the adapter to a computer. An aluminum frame provides the mechanical stability for the board, as well as electromagnetic shielding. The RHD2000 (Intan Technologies LLC, Los Angeles, CA, USA) software controls the measurement.

Figure 3a depicts the clamped chip in the measurement adapter and Figure 3b a cross section of the set-up. All measurements use buffered saline (PBS) as an electrolyte in a two-electrode setup, where the thick film gold serves as working electrode and a platinum wire as counter/reference electrode. The measurements were carried out at 20–5000 Hz. The signal characteristic of neuronal spikes requires an excellent signal coupling at frequencies around 1 kHz. This frequency is therefore used in many works as a reference value for impedance measurements. Comparative measurements were thus carried out at this frequency. Statistical analyses were realized using the software MiniTab17.

## 3. Results and Discussion

### 3.1. Influence of Electrode Size

The influence of the electrode size was investigated using MEA type B. Uncoated thick film gold electrodes with varying size between 86 µm and 215 µm are arranged on these chips. The impedance measurement was carried out after cleaning. The box plot of the impedance magnitude measured at 1 kHz is depicted as a function of the diameter in Figure 4.

As expected, the impedance magnitude increases with decreasing diameter. The respective impedance per unit area varies strongly, in particular for small electrodes. It increases by a factor of 8 for an electrode diameter of 86 µm when compared to electrodes with a diameter of 129 µm, which corresponds to a surface area ratio of 2.25. The variation of the impedance magnitude also increases significantly if the electrodes become smaller. The fluctuating signal transmission on small electrodes is therefore a source of measurement uncertainty. Pronounced boundary effects due to field inhomogeneities are assumed to be the reason for this characteristic.

### 3.2. Influence of the Laminin Coating and Repeated Applications

Type C chips prepared pursuant to the laminin coating protocol and filled with 10% DMEM were used. The impedance measurement was carried out after one day of dwell time at 1 kHz. After the measurement, the chips were cleaned using tergazyme and coated again, repeating the procedure four times (cycles 1–4). No leakage was observed in this test. The results in Figure 5 demonstrate that the coating significantly reduces the impedance magnitude. In comparison with the uncoated electrodes, the impedance magnitude decreases by one half or more. In cycle 2, a significant deviation is observed, which is assumed to be a consequence of the manual coating process; the layer thickness can vary, dependent on individual handling. However, repeated application and coating does not impair the performance. The values at cycle 4 are the lowest in this trial. The respective measurement was carried out after 7 days’ dwell time. The influence of the electrode size on the impedance was significantly reduced. The impedance per unit area of the smaller electrodes was still higher, but the factor was less than three.

### 3.3. Influence of Functionalization

Comparison of the impedance characteristic for small electrodes (86 µm diameter) with different functionalization shows that coatings can significantly improve the impedance characteristic.

The low-frequency impedance response of the gold electrolyte system is predominantly capacitive; it can be described by a simple serial equivalent circuit, consisting of a serial resistance Rs and serial capacitance Cs. Figure 6 illustrates that thick film gold electrodes are characterized by low values for Cs (0.35 pF @ 1 kHz) and high values for Rs (117 kΩ @ 1 kHz). The Nyquist plot in Figure 7 can be approximated by a circle function. This characteristic entails a high damping of low-frequency signals.

To describe the behavior of the coated electrodes, charge transfer and diffusion components must be added. The equivalent circuit in Figure 7 proposes an additional RC element for functionalized electrodes. In the case of pure gold, the double layer capacitance is the limiting factor for the signal transition. Functionalization changes zeta potential and charge transfer, resulting in significantly different characteristics. TiO_x_N_y_ functionalization generates a mixed kinetic and charge-transfer characteristic, with a pronounced diffusion dominated range between 200 Hz and 1 kHz, which is apparent through the straight line with a slope of approximately 45° in the Nyquist plot. At high frequencies, low serial capacitance and serial resistance are related. The serial resistance at 5 kHz amounts to 20 kΩ. The capacitance of the functionalization itself is assumed to be high because of the very low TiO_x_N_y_ layer thickness of 25 nm and high permittivity of the material. PEDOT:PSS functionalization leads to mixed characteristic of the transmission too. The layer thickness is in the range of a few hundred nanometer. The layer has a higher serial resistance in comparison to TiO_x_N_y_ coating, and the serial capacitance is significantly lower.

## 4. Conclusions

Thick film gold electrodes are characterized by high impedance magnitude and deviation. The dominating influence of the double layer capacitance can explain this behavior. Very strong variance of the impedance per unit area dependent on electrode diameter is observed. This leads to high measurement uncertainty, especially for small electrodes, which are desirable for the monitoring of neuronal cell cultures. Laminin coating weakens this effect. The electrodes can be recycled for four cycles without effecting the performance, but variation in the manual coating procedure can cause significant differences.

The high double layer barrier of thick film gold electrodes is decreased by functionalization layers. PEDOT:PSS layers applied by doctor blade and sputtered TiO_x_N_y_ layers have been evaluated. Both functionalization methods lead to a mixed kinetic and charge-transfer characteristic. The best signal transition properties are expected when TiO_x_N_y_ functionalization is used. Small electrodes with 86 µm diameter functionalized with these layers have a high serial capacitance of 4.1 pF and low serial resistance of 32 kΩ at a frequency of 1 kHz. These electrodes should therefore be suitable for signal capture in neuronal cell cultures in vitro.

Functionalized thick film gold electrodes arranged on miniaturized ceramic contact boards made of LTCC can be integrated in bio-reactors with temperature control and flow monitoring in the future.

## Figures and Tables

**Table 1:** Design and electrode geometry of different MEA types.

Type A	Type B	Type C
Array of 60 electrodes Diameter 86 µm Pitch 200 µm	Array with different electrode sizes: Column 1 and 8: 6 × 86 µm Column 2 and 7: 8 × 129 µm Column 3 and 6: 8 × 172 µm Column 4 and 5: 8 × 215 µm Pitch Column 1–2, 2–3, 6–7, 7–8: 546 µm Pitch Column 3–4, 4–5, 5–6: 893 µm Rows pitch is equivalent	3D MEA: 27 tower electrodes on MCCB: Diameter 86 µm Pitch in z-direction 150 µm Finger distance 500 µm 30 base plate electrodes Diameter 129 µm Pitch (x-y): 425 µm, Diagonal distance: 600 µm
Functionalization		
TiOxNy and PEDOT:PSS	Without	Laminin coating
Related to results in section		
3.3	3.1	3.1/3.2/3.3

**Table 2:** Variation of application parameters for PEDOT:PSS.

Chip N°	Blade Gap [µm]	Wetting Agent [%]	Drying Temperature [°C]	Mg Imp @ 1 kHz [kΩ]	Phase Angle @ 1 kHz [°]	R_s_ @ 1 kHz MW [kΩ]	C_s_ [pF]
22	150	no	65°	81	54.5	37	2.5
23	300	0.01	65°	281	67	55	1.0
24	300	0.01	75°	333	70	53	0.9

## Supplementary Materials

Supplementary File 1
